# Impact of Repetitive Transcranial Magnetic Stimulation on Neurocognition and Oxidative Stress in Relapsing-Remitting Multiple Sclerosis: A Case Report

**DOI:** 10.3389/fneur.2020.00817

**Published:** 2020-08-07

**Authors:** Eduardo Agüera, Javier Caballero-Villarraso, Montserrat Feijóo, Begoña M. Escribano, María C. Bahamonde, Cristina Conde, Alberto Galván, Isaac Túnez

**Affiliations:** ^1^Instituto Maimónides de Investigación Biomédica de Córdoba (IMIBIC), Córdoba, Spain; ^2^Unidad de Gestión Clínica de Neurología, Hospital Universitario Reina Sofía, Córdoba, Spain; ^3^Departmento de Bioquímica y Biología Molecular, Facultad de Medicina y Enfermería, Universidad de Córdoba, Córdoba, Spain; ^4^Unidad de Gestión Clínica de Análisis Clínicos, Hospital Universitario Reina Sofía, Córdoba, Spain; ^5^Departamento de Biología Celular, Fisiología e Inmunología, Universidad de Córdoba, Córdoba, Spain

**Keywords:** compassionate use trial, multiple sclerosis, disability, neuroplasticity, oxidative stress, psychometry, transcranial magnetic stimulation, case report

## Abstract

Multiple sclerosis (MS) is a neurodegenerative condition whose manifestation and clinical evolution can present themselves in very different ways. Analogously, its treatment has to be personalized and the patient's response may be idiosyncratic. At this moment there is no cure for it, in addition to its clinical course sometimes being torpid, with a poor response to any treatment. However, Transcranial Magnetic Stimulation (TMS) has demonstrated its usefulness as a non-invasive therapeutic tool for the treatment of some psychiatric and neurodegenerative diseases. Some studies show that the application of rTMS implies improvement in patients with MS at various levels, but the effects at the psychometric level and the redox profile in blood have never been studied before, despite the fact that both aspects have been related to the severity of MS and its evolution. Here we present the case of a woman diagnosed with relapsing-remitting multiple sclerosis (RRMS) at the age of 33, with a rapid progression of her illness and a poor response to different treatments previously prescribed for 9 years. In view of the patient's clinical course, a compassionate treatment with rTMS for 1 year was proposed. Starting from the fourth month of treatment, when reviewing the status of her disease, the patient denoted a clear improvement at different levels. There followed out psychometric evaluations and blood analyses, that showed both an improvement in her neuropsychological functions and a reduction in oxidative stress in plasma, in correspondence with therTMS treatment.

## Introduction

The clinical manifestations of multiple sclerosis (MS) can be highly varied, both in its onset and insubsequent outbreaks. It can present itself with astenia, muscle weakness, ataxia, spasticity, dysarthria and dysphagia, among other symptoms, and signs. Depending on its severity, MS can limit the daily activities of patients and end up producing a relevant degree of disability. Hence, it is the main reason for non-traumatic, invalidating neurological disease in young adults, and is the principal cause of disability acquired between 20 and 40 years of age (1, 2).

Given the heterogeneity of the forms and manifestations of MS, its treatment has to be individualized and there are diverse drugs available within the therapeutic arsenal. In spite of this, on many occasions the disease advances inexorably (2, 3).

However, one alleviative possibility may be transcranial magnetic stimulation (TMS), a non-invasive tool that activates the brain, based on the principle of electromagnetic induction. According to this premise, a current passing through a coil placed on the scalp of an individual may induce a magnetic field that reaches the brain surface with an insignificant attenuation. This magnetic field is, in turn, capable of generating a secondary ionic current, that depolarizes the neurons. The standard device for TMS is fairly simple and has an adaptive use; consequently, several parameters can be changed according to the purpose of the stimulation, such as its application site, intensity and frequency, duration, and length of the therapy. The modulation of the frequency of the repetitive pulses of TMS (rTMS) permits the obtaining of notably different changes in the excitability of the area stimulated, while the treatments signify a longer duration of the effects (4, 5).

This peculiar therapeutic resource was authorized in 2008 for the treatment of refractory major depression by the Food and Drug Administration (FDA) and later by the European Medicines Agency (EMEA). In recent years, research has revealed the therapeutic potential of TMS in the treatment of Alzheimer's disease, improving the psychomotor skills and memory of affected patients. Similarly, the application of TMS in Parkinson's patients has been observed to lead to symptomatic improvement at different levels, such as decreased essential tremor, spastic rigidity, and bradykinesia. Furthermore, TMS has shown a clear effectiveness in reducing neuropathic pain and in the degree of spasticity subsequent to an ischemic stroke (6). Analogously to other neurodegenerative diseases, TMS has been used in MS, showing very satisfactory results in terms of spasticity, fatigue, cognition, pain, sensory defects, lower urinary tract dysfunction and dysphagia (6–8).

We present a case of relapsing-remitting MS in which, 7 years after its onset and a torpid evolution, a compassionate use of rTMS was authorized. The patient was a woman diagnosed at 33 years of age with multiple sclerosis, clinically defined as being the relapsing-remitting type, according to the revised McDonald criteria of 2005 in force on that date (in 2008) from 2 clinical outbreaks that had taken place in previous years (8). It is the first time that the scientific literature deals with the joint study of neurocognition and oxidative stress in blood to check the effects of TMS on MS.

## Case Presentation

A 33 year old woman who at the end of 2002 went to the emergency department complaining of insomnia and fatigue. She was diagnosed with anxiety disorder and was discharged. Six weeks later she was readmitted to the hospital with weakness, generalized myalgia, and a tingling sensation in her extremities. Given the early recurrence, the neurology department was consulted. In the neurological exam her mental state was normal, her pupils were normal symmetrical, and reactive to light. There was no facial asymmetry and the other cranial nerves were normal. Muscle tone was increased in bilateral lower extremities. Grade II-III spasticity was diagnosed, as well as decreased vibratory sensitivity and bilateral Hoffman sign (involuntary flexion movement of the index finger when the examiner flicks the fingernail of the middle finger down), bilateral Babinski sign (plantar flexor response) and positive Romberg sign, with a tendency to fall to the right. Routine blood test was normal, except for cholesterol level (268 mg/dL). Magnetic resonance imaging (MRI) detected seven of demyelinating lesions of various sizes, especially periventricular and supratentorial and some infratentorial, as well as cortico-subcortical atrophy. No signs of progressive multifocal leukoencephalopathy were visualized.

In mid- 2003 she had an optical neuritis (left retrobulbar type) visual outbreak, with a complete recovery, and 4 years later a spinal outbreak that presented itself as a paraparesis.

The patient began treatment with β-1a interferonin 2008 and because of her lack of response to the treatment due to some outbreaks and an increase in EDSS of up to a score of 5.0, in 2009 it was considered convenient to change to Natalizumab as a high activity treatment. She continued that treatment for 7 years but the persistence of attacks, although spread over the time, and the progression of associated disability (EDSS score 5.5), indicated a further change to another high efficiency therapy, i.e., Alemtuzumab, with which she completed the two cycles indicated on the data sheet. During her neurological examination the prominent features were: a visual optical neuritis-type aftermath, a minimal oculomotor alteration, invalidating spastic paraparesis, right hemiparesis, and sphincter disorders which currently would be defined by a score of 6.0 on the EDSS scale.

Standing out as comorbidities were: depression as a reaction to her illness, notable spasticity, fatigue and joint pain, all of them being treated pharmacologically for the past 5 years.

Three years ago, given her disability and lack of response to the symptomatic treatment prescribed, rTMS treatment was proposed, which she accepted. Based on the results obtained with TMS in previous clinical studies carried out in patients with MS, as well as in its equivalent experimental model in rats (experimental autoimmune encephalomyelitis, EAE) and in other neurodegenerative diseases, the main objective was to improve the degree of disability in this patient (6, 9–16). We also wanted to examine her evolution at the neuropsychological level and her biomarkers of oxidative damage in blood, in order to look for links between these aspects (17–19).

### Laboratory Investigations and Diagnosis Tests

Cycles of a daily session of rTMS were prescribed. These were grouped into 5 consecutive days, followed by 3 weeks resting, until 14 cycles (70 sessions in all) were completed, spread over 14 months. The stimulation was applied at 3 cm in front of the Cz point in the craniocaudal axis. The stimulation power was established as a function of the motor threshold of the patient, representing 90% of its total value. It was administered as a continuous stimulation with a frequency of 1 Hz during a span of 600 s in each session.

In each session, we follow this protocol in two steps:

First step: Obtaining the Motor Evoked Threshold at Rest: the patient had her threshold evoked motor calculated at rest, by stimulation of the motor cortex in the left hemisphere, evoking electromyographic responses (EMG) in the contralateral muscles, called motor evoked potentials (MEP). The threshold of motor excitability at rest (TM) is defined as the minimum intensity (expressed as the percentage of the maximum output power of the stimulator) capable of producing a reproducible MEP in a resting muscle in 50% of 10 shots.
Second step: Administration of the Transcranial Magnetic Stimulation: A Rapid 2 Magstim device (Magstim Co.®, Whitland, Carmarthenshire, Wales) equipped and connected to a figure-of-eight coil of 70 mm was used. This equipment is used to calculate the TM and the percentage of it to which the treatment will have to be applied. The selection of the specific point of stimulation in the Supplementary Motor Area (SMA) was sufficiently anterior to prevent the propagation of the impulse from triggering the muscular contraction of the shoulders, trunk, and lower limbs. The position of the coil was marked on the scalp to ensure consistent placement of the coil throughout the experiments; the patient was fitted with a lycra cap on which to indicate and also mark the exact stimulation point, as well as on which to place and hold the coil during the therapeutic session. The coil was oriented toward the posterior area in order to trigger a postero-anterior current. The TM was calculated in relation to the evoked potential and according to international standards. Based on it, TM was defined as the lowest stimulus intensity that elicited a minimum MEP amplitude of 50 mV in the completely relaxed FDI muscle in at least 5 out of 10 consecutive trials (20, 21).

**Figure 1:**
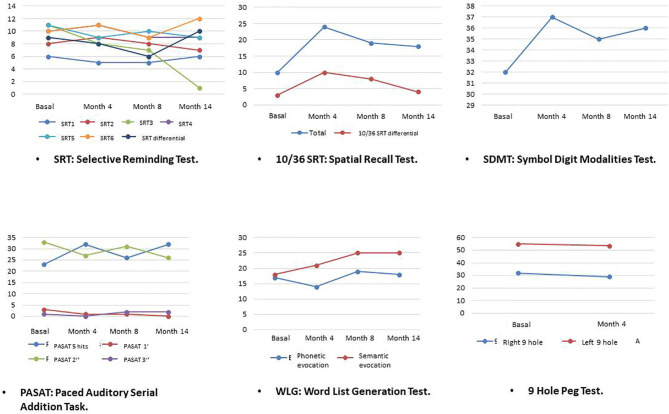
Evolution of psychometric scores.

**Figure 2:**
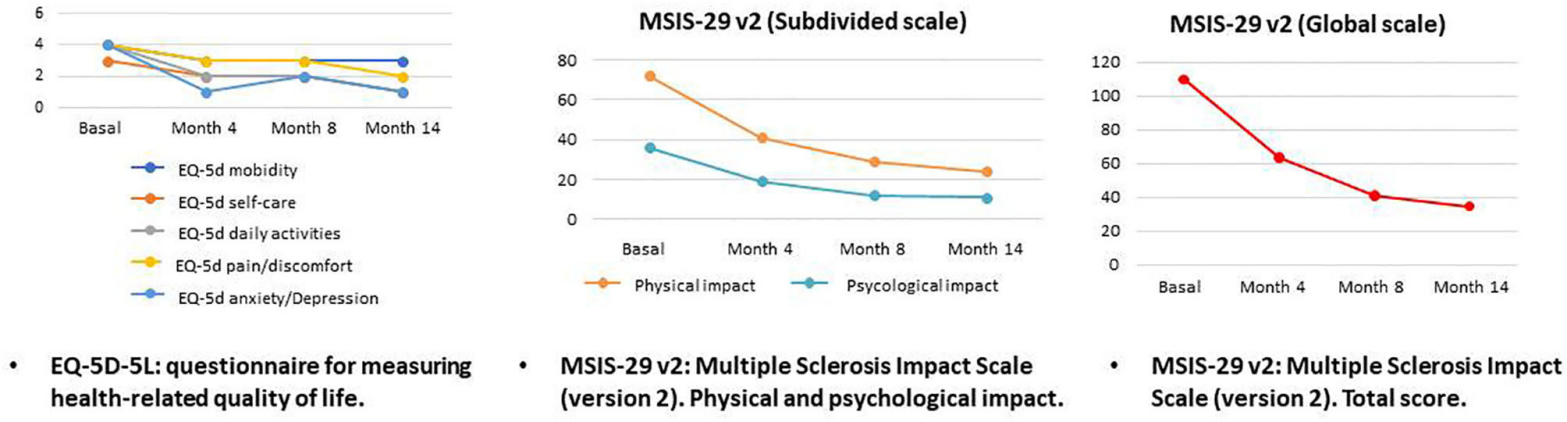
Evolution of self-perception scores.

During this treatment time, periodic physical and neurocognitive evaluations were made, as well as blood analyses (Table 1). The neuropsychological tests were generally orientated toward assessing the patient's cerebellar and motor functions (as in the 9 HolePeg Test and the Timed 25-Foot Walk) and her short and medium term mnesic capacity (as is the case of the Selective Reminding Test, 10/36 Spatial Recall Test, Symbol DigitModalities Test, Paced Auditory Serial AdditionTask and Word List Generation Test). Generally speaking, the results of the tests showed an improvement in the patient's motor and neurocognitive abilities (displaying an increase in the scoring scales), as well as a lower degree of disability (falling from 6 to 5 points in the EDSS scale as from the second month of treatment with rTMS) (Figure 1) and a better perception of her health in relation to the impact of the disease on it (showing a drop in the EQ-5D-5L and MSIS-29 scales scoring) (Figure 2). This improvement, according to the evaluation scales, was produced practically starting from a short time after the beginning of the treatment (at between 4 and 8 months), and was maintained the whole time. Only a small increase in Timed 25-Foot Walk was observed; in the first four evaluations it lasted 12 s and in the last one 15, which could correspond to a slight slowing down of her ambulation.

**Table 1:** Schedule of cycles and performed tests.

**Cycle number**	0 (^*^)	**1**	**2**	**3**	**4**	**5**	**6**	**7**	**8**	**9**	**10**	**11**	**12**	**13**	**14**
Studied items	EDSS EQ-5D-5L MSIS-29 SRT 10/36 SRT SDMT PASAT WLG 9 hole T25-FW				EDSS EQ-5D-5L MSIS-29 SRT 10/36 SRT SDMT PASAT WLG 9 hole T25-FW				EDSS EQ-5D-5L MSIS-29 SRT 10/36 SRT SDMT PASAT WLG 9 hole T25-FW						EDSS EQ-5D-5L MSIS-29 SRT 10/36 SRT SDMT PASAT WLG 9 hole T25-FW
									Blood test						Blood test

* *Baseline assessment, one more before starting rTMS*.

*EDSS, Expanded Disability Status Scale; EQ-5D-5L, questionnaire for measuring health-related quality of life; MSIS-29, Multiple Sclerosis Impact Scale (version 2); SRT, Selective Reminding Test; 10/36 SRT, Spatial Recall Test; SDMT, Symbol Digit Modalities Test; PASAT, Paced Auditory Serial Addition Task; WLG, Word List Generation Test; 9 hole, Nine-Hole Peg Test, to measure finger dexterity; T25-FW, Timed 25-Foot Walk*.

The laboratory study took into consideration routine biochemical magnitudes and oxidative stress parameters (Table 2). It was carried out at two moments: half-way through and at the end of the treatment period. In both laboratory studies a very slight, or clinically irrelevant variation was generally observed, although the oxidative stress parameters displayed a notable change, with a tendency toward an antioxidant status.

**Table 2:** Laboratory tests in plasma.

	**Parameter**	**Month 8th**	**Mont 14th**
**Routine biochemistry**			
	Glucose (mg/dl)	94	87
	Total cholesterol (mg/dl)	230	229
	c-HDL (mg/dl)	62	60
	c-LDL (mg/dl)	149	143
	Triglycerides (mg/dl)	95	130
	BUN (mg/dl)	30	18
	Total proteins (g/dl)	7.2	6.6
	Albumin (g/dl)	4.5	4.2
	Ceruloplasmin (ng/dl)	28	27
	Transferrin (mg/dl)	304	298
	Creatinine (mg/dl)	0.65	0.60
	Total Bilirubin (mg/dl)	0.7	0.5
	Uric acid (mg/dl)	3.8	3.6
	Aspartate transferase (AST: U/l)	17	16
	Alanine transferase (ALT: U/l)	15	14
	Gamma glutamil transpeptidase (γGT: U/l)	17	19
	Alkaline phosphatase (U/l)	41	42
	Angiotensin-converting enzyme (ACE: μg/l)	29	25
**Oxidative stress biomarkers**			
	Lipid Peroxides products (LPO: μM/ml)	2.11	1.97
	Carbonilated proteins (CP: nM)	0.37	0.25
	Total glutathione (tGSH: μM/ml)	0.55	0.60
	Reduced glutathione (GSH: μM/ml)	0.09	0.12
	Oxidized glutathione (GSSG: μM/ml)	0.46	0.48
	GSH/GSSG ratio	0.20	0.25

## Discussion

Some authors had previously reported on the benefits of rTMS in patients with MS. On studying them, it was basically demonstrated that administering rTMS on the motor cortex could have an advantageous effect on the spasticity of these patients as it improved the excitability of the corticospinal tract and reduced the stretch reflex (8, 10–14). The actual mechanism by which this spasticity attenuation is produced is unknown. However, if it is taken into account that corticospinal cells modulate the activity of the afferent alpha and gamma motorneurons and a large group of spinal interneurons, the modulation of the motor cortex excitability by rTMS could result in the triggering of a significant modulation of the spinal excitability. On this basis, it could be argued that the beneficial effects observed on spasticitycould be related to plastic changes in the spinal circuits, induced throughout the repetitive TMS (rTMS) (22, 23). There are several possible mechanisms to explain the therapeutic effects of rTMS. For example, the cortical magnetic stimulation induces long term potentiation-like effects at the synapses in the neuronal circuitry of the motor cortex via the glutamatergic neuron or GABAB autoreceptor activation. This type of stimulation elevates the expression of the brain-derived neurotrophic factor, nerve growth factor and neuromodulators, such as cholecystokinin, which influence synaptic plasticity and neuron survival. Therefore, rTMS can enhance the cortical synaptic plasticity and increased synaptic plasticity as a compensatory mechanism for the incomplete remyelination (15, 24).

As well as on spasticity, TMS could act on the evolution of MS by influencing other symptoms of this disease. Several authors have used this technique in their studies on fatigue, a common and debilitating symptom of MS, whose physiopathological origin is still not understood (25–28).

The etiopathogenic mechanisms responsible for neuropsychological disorders associated with MS are not well-known either. What makes the case presented here precisely a special one is that the evolution of these conditions was evaluated after rTMS administration. The psychometric evaluations were conducted by a neuropsychologist and, as described above, tests and validated scales were employed for the rating of neurocognitive skills related to the functioning of the brain cortex. This case is also distinctive for having studied the evolution of the biochemical profile.

It is of interest to observe that practically all the neuropsychological characteristics evaluated in this patient exhibited an improvement that was maintained throughout the treatment. The Timed 25-Foot Walk test was the only one that did not show this improvement so clearly. In fact, in the last evaluation, the time elapsed in taking the 25 steps increased from 12 to 15 s; however, it might be thought that this increase in time could be due to the length of the stride being longer and, actually, the patient's walk became more functional and effective.

After a year of finishing the rTMS prescribed, the patient did not wish to return as she felt better. Consequently, we were not able to report the time during which the effects were maintained. We logically need to be cautious in the interpretation of all these findings. To begin with, any improvement in the intellectual or emotional sphere could be susceptible to a placebo effect. However, the correlation of the clinical improvement with the biochemical changes could point to the objectivity of the results observed.

Our observations in the case of this patient endorse the fact that in the etiopathogeny of MS, in addition to inflammatory processes, oxidative stress mechanisms are involved and that the seriousness of the disease is also associated with the intensity of that stress (29–31). It would similarly confirm that rTMS therapy would exert an objectifiable clinical improvement on MS and that such improvement would, in turn be related to a diminution in oxidative stress. Over the past few years, numerous experimental studies have been performed on the effects of TMS on Experimental Autoimmune Encephalitis (EAE), which reproduces a multiple sclerosis (MS-like) experimental model. In these studies it has been observed that, besides improving the clinical score of the disease, TMS treatment is related to an improvement in the molecular profile of the individuals treated (less oxidative stress and inflammation) (17–19), and even to a greater brain cell density (32). Therefore, rTMS administration may have an antioxidant effect, which would be responsible for the clinical improvement alluded to.

Regarding the weaknesses and strengths of this case, we are aware that more parameters could have been examined in the patient. However, we did not want to disturb her excessively since the rTMS sessions are already long and we had to add to this the time of the neuropsychological evaluations (given that they were not included in the standar service portfolio). That is why we focused on tests that were less studied in the scientific literature. Only Azin et al. described the neuropsychological facet on MS after rTMS treatment in a study of 36 patients (19 under rTMS and 17 sham group). Similar to this case report, Azin's group observed an improvement in patients' manual dexterity after treatment. They studied the ‘Nine-Hole Peg Test,’ but the rest of the psychological items that we present are novel (16). There are also no studies in the literature that have checked the biochemical profile about oxidative damage in MS patients after being treated with TMS. Our group reported an improvement in the oxidative profile in the experimental autoimmune encephalomyelitis model (17–19). Given that we had a limited blood sample volume, we had to select the parameters to analyze and we chose to select those biochemical magnitudes of oxidative stress in which we had the most experience.

What can be considered as a strength of this case report is the safety of long-term rTMS treatment. Throughout all the sessions, there were no complications or adverse effects in this patient, which may be due to rTMS. Other research groups also agree on these observations (8, 33–35).

## Conclusion

The clinical improvement in the evolution of this patient after being treated with rTMS suggests that this therapy is effective in MS. This is in tune with the results observed after administering rTMS in other neurological and psychiatric conditions that are in a certain way analagous to MS (with respect to its etiology, pathogeny, or clinical manifestations). It is also in line with the results observed in studies made in experimental models. The clinical improvement could also be associated with the decline in the oxidative stress observed after rTMS administration. These facts make it recommendable to carry out clinical trials with exhaustive and prolonged monitoring in order to examine the physical, neuropsychological, and molecular effects of TMS on patients with MS, as well as the time over which these effects are maintained.

## Data Availability Statement

All datasets generated for this study are included in the article/Supplementary Material.

## Ethics Statement

Written informed consent was obtained for the publication of this case report.

## Author Contributions

MB, CC, and AG carried out the clinical evaluations and collected the clinical information. MF and BE performed the laboratory study. EA and JC-V analyzed all the data and drafted the manuscript. IT designed and supervised the studies. All authors approved the final version of the manuscript.

## Conflict of Interest

The authors declare that the research was conducted in the absence of any commercial or financial relationships that could be construed as a potential conflict of interest.
