# Supplementary material for: Impact of Repetitive Transcranial Magnetic Stimulation on Neurocognition and Oxidative Stress in Relapsing-Remitting Multiple Sclerosis: A Case Report
Source: Front Neurol. 2020 Aug 7;11:817. doi: 10.3389/fneur.2020.00817 (PMC7438891; doi:10.3389/fneur.2020.00817)
Supplement: Supplementary file 1 [file Data_Sheet_1.PDF]

FORMULARIO DE INFORMACIÓN Y CONSENTIMIENTO INFORMADO ESCRITO

Orden de 8 de julio de 2009 (BOJA nº 152 de fecha 6 de agosto) por la que se dictan instrucciones a los Centros del Sistema Sanitario Público de Andalucía, en relación al procedimiento de Consentimiento Informado.

CENTRO SANITARIO<br>HOSPITAL UNIVERSITARIO REINA SOFÍA	SERVICIO DE<br>NEUROLOGÍA

1 DOCUMENTO DE INFORMACIÓN PARA ESTIMULACION MAGNETICA TRASNCRANEAL

Este documento sirve para que usted, o quien lo represente, dé su consentimiento para esta intervención. Eso significa que nos autoriza a realizarla.

Puede usted retirar este consentimiento cuando lo desee. Firmarlo no le obliga a usted a hacerse la intervención. De su rechazo no se derivará ninguna consecuencia adversa respecto a la calidad del resto de la atención recibida. Antes de firmar, es importante que lea despacio la información siguiente.

Díganos si tiene alguna duda o necesita más información. Le atenderemos con mucho gusto.

(*) Indicar el nombre del procedimiento/intervención a realizar; si es posible, además del nombre técnico que siempre debe figurar, puede tratar de expresarlo con un nombre más sencillo.

1.1 LO QUE USTED DEBE SABER:

EN QUÉ CONSISTE. PARA QUÉ SIRVE:

Los investigadores utilizan la TMS para conocer cómo funciona el cerebro. Por ejemplo, la TMS ayuda a los investigadores a conocer cómo la actividad cerebral controla el comportamiento y cómo se organiza el cerebro. Los investigadores también utilizan la TMS para comprender cómo la actividad cerebral es diferente en pacientes con distintas enfermedades o afecciones. Estas son algunas de las enfermedades y afecciones: Enfermedades en que se se usa: enfermedad de Parkinson enfermedad de Alzheimer epilepsia esquizofrenia autismo lesión cerebral traumática dolor crónico accidente cerebrovascular. La Estimulación Magnética Transcraneal (EMT) es una técnica no invasiva que comenzó a utilizarse hace más de veinte años y ha demostrado su eficacia en el tratamiento de algunos trastornos mentales, como por ejemplo la depresión, trastorno de ansiedad generalizada, trastorno de estrés postraumático, ataques de pánico y en otras patologías.

CÓMO SE REALIZA:

Puede utilizar un gorra de natación o una cinta para la cabeza mientras el investigador sostiene la “bobina” de TMS sobre la cabeza. Luego, el investigador iniciará la estimulación. Esto crea una energía magnética llamada “pulso”. El pulso genera una corriente que estimula una pequeña parte del cerebro. Usted sabrá cuándo la bobina se encuentra sobre su cabeza porque escuchará un sonido de clic y puede sentir pequeñas presiones en la cabeza. Es posible que los investigadores también utilicen electrodos (pequeños discos con cables) para medir los efectos de la estimulación. Normalmente, la TMS se considera segura y relativamente indolora.

QUÉ EFECTOS LE PRODUCIRÁ:

El efecto secundario más común es un dolor de cabeza leve. Esto puede suceder durante la TMS o justo despúés de esta. Dependiendo de dónde los investigadores ubican la bobina, es posible que los músculos de la cara, la cabeza, el cuello y las manos se contraigan o muevan ligeramente. Esto puede ser incómodo. El sonido de clic que proviene de la TMS puede afectar la audición durante un tiempo breve. Los investigadores le darán tapones para los oídos para ayudarlo a proteger la audición. Existe un leve riesgo de que la TMS le produzca convulsiones, pero esto es muy raro. Es posible que existan otros efectos secundarios debido a la TMS que los investigadores todavía no conocen.

EN QUÉ LE BENEFICIARÁ:

0 0 1 5 3 0

Su mecanismo de acción consiste en estimular directamente regiones de la corteza cerebral, como la corteza prefrontal y otras áreas asociadas con la regulación del estado de ánimo; mediante el uso de la energía eléctrica que pasa a través de una bobina de estimulación para generar un campo magnético potente. Durante una sesión de tratamiento, la energía de este campo magnético se transfiere en el cerebro de un paciente cuando se aplica una bobina sobre la cabeza de una persona. La energía magnética pasa fácilmente a través de la piel y el cráneo, la activación cerebral es indolora y no necesita sedación. De forma simplificada se puede decir que la estimulación magnética transcraneal produce un aumento o disminución de la actividad neuronal, además cambios en el metabolismo de algunos neurotransmisores como GABA y serotonina en algunas áreas cerebrales.

OTRAS ALTERNATIVAS DISPONIBLES EN SU CASO:

001530

CENTRO SANITARIO<br>HOSPITAL UNIVERSITARIO REINA SOFÍA	SERVICIO DE<br>NEUROLOGÍA

QUÉ RIESGOS TIENE:

Cualquier actuación médica tiene riesgos. La mayor parte de las veces los riesgos no se materializan, y la intervención no produce daños o efectos secundarios indeseables. Pero a veces no es así. Por eso es importante que usted conozca los riesgos que pueden aparecer en este proceso o intervención.

- LOS MÁS FRECUENTES:

- LOS MÁS GRAVES:

- LOS DERIVADOS DE SUS PROBLEMAS DE SALUD:

SITUACIONES ESPECIALES QUE DEBEN SER TENIDAS EN CUENTA:

OTRAS INFORMACIONES DE INTERÉS (a considerar por el/la profesional):

Si requiere información adicional se puede poner en contacto con nuestro personal del Servicio de Neurologota, Hospital Universitario Reina Sofía o del Dpto. de Bioquímica y Biología Molecular de la Facultad de Medicina, IMIBIC/Universidad de Córdoba en el teléfono: 957 218 268 o 957 012 352 o en el correo electrónico: fm2tufii@uco.es o fernansanzlo@hotmail.com

OTRAS CUESTIONES PARA LAS QUE LE PEDIMOS SU CONSENTIMIENTO:

- A veces, durante la intervención, se producen hallazgos imprevistos. Pueden obligar a tener que modificar la forma de hacer la intervención y utilizar variantes de la misma no contempladas inicialmente.

- A veces es necesario tomar muestras biológicas para estudiar mejor su caso. Pueden ser conservadas y utilizadas posteriormente para realizar investigaciones relacionadas con la enfermedad que usted padece. No se usaran directamente para fines comerciales. Si fueran a ser utilizadas para otros fines distintos se le pediría posteriormente el consentimiento expreso para ello. Si no da su consentimiento para ser utilizadas en investigación, las muestras se destruirán una vez dejen de ser útiles para documentar su caso, según las normas del centro. En cualquier caso, se protegerá adecuadamente la confidencialidad en todo momento.

- También puede hacer falta tomar imágenes, como fotos o videos. Sirven para documentar mejor el caso. También pueden usarse para fines docentes de difusión del conocimiento científico. En cualquier caso serán usadas si usted da su autorización. Su identidad siempre será preservada de forma confidencial.

001530

CENTRO SANITARIO<br>HOSPITAL UNIVERSITARIO REINA SOFÍA	SERVICIO DE<br>NEUROLOGÍA
**1.2 IMÁGENES EXPLICATIVAS**	
En este espacio podrán insertarse con carácter opcional imágenes explicativas, esquemas anatómicos, pictogramas etc. que faciliten y permitan explicar de manera más sencilla la información al paciente.	

001530

CENTRO SANITARIO<br>HOSPITAL UNIVERSITARIO REINA SOFÍA	SERVICIO DE<br>NEUROLOGIA

2 CONSENTIMIENTO INFORMADO

2.1 DATOS DEL/DE LA PACIENTE Y DE SU REPRESENTANTE (sólo en caso de incapacidad del/de la paciente)

APELLIDOS Y NOMBRE, DEL PACIENTE	DNI / NIE
ABAD BRIEGA ANTONIA	44355817 H
APELLIDOS Y NOMBRE, DEL/DE LA REPRESENTANTE LEGAL	DNI / NIE

2.2 PROFESIONALES QUE INTERVIENEN EN EL PROCESO DE INFORMACIÓN Y/O CONSENTIMIENTO

APELLIDOS Y NOMBRE	FECHA	FIRMA
BAHAMONDE ROMAN, CARMEN	6/JUN/2016	[firma]
APELLIDOS Y NOMBRE	FECHA	FIRMA
APELLIDOS Y NOMBRE	FECHA	FIRMA
APELLIDOS Y NOMBRE	FECHA	FIRMA
APELLIDOS Y NOMBRE	FECHA	FIRMA

2.3 CONSENTIMIENTO

Yo, D/Dña Antonia Abad Briega, manifiesto que estoy conforme con la intervención que se me ha propuesto. He leido y comprendido la información anterior. He podido preguntar y aclarar todas mis dudas. Por eso he tomado consciente y libremente la decisión de autorizarla. También sé que puedo retirar mi consentimiento cuando lo estime oportuno.

☒ SI ☐ NO Autorizo a que se realicen las actuaciones oportunas, incluyendo modificaciones en la forma de realizar la intervención, para evitar los peligros o daños potenciales para la vida o la salud, que pudieran surgir en el curso de la intervención.

☒ SI ☐ NO Autorizo la conservación y utilización posterior de mis muestras biológicas para investigación relacionada directamente con la enfermedad que padezco.

☒ SI ☐ NO Autorizo que, en caso de que mis muestras biológicas vayan a ser utilizadas en otras investigaciones diferentes, los investigadores se pongan en contacto conmigo para solicitarme consentimiento.

☒ SI ☐ NO Autorizo la utilización de imágenes con fines docentes o de difusión del conocimiento científico.

NOTA: Márquese con una cruz.

En Córdoba a 6 de Junio de 2016

EL/LA PACIENTE EL/LA REPRESENTANTE LEGAL (sólo en caso de incapacidad del paciente)

Fdo.: Antonia Abad Briega Fdo.:

CENTRO SANITARIO<br>HOSPITAL UNIVERSITARIO REINA SOFÍA	SERVICIO DE<br>NEUROLOGÍA

001530

2.4	RECHAZO DE LA INTERVENCIÓN

Yo, D/Dña. , no autorizo a la realización de esta intervención. Asumo las consecuencias que de ello puedan derivarse para la salud o la vida.

En a de de

EL/LA PACIENTE

EL/LA REPRESENTANTE LEGAL (sólo en caso de incapacidad del paciente)

Fdo.:

Fdo.:

2.5	REVOCACIÓN DEL CONSENTIMIENTO

Yo, D/Dña , de forma libre y consciente he decidido retirar el consentimiento para esta intervención. Asumo las consecuencias que de ello puedan derivarse para la salud o la vida.

En a de de

EL/LA PACIENTE

EL/LA REPRESENTANTE LEGAL (sólo en caso de incapacidad del paciente)

Fdo.:

Fdo.:

001530
